# Commentary on “Simple non-invasive biomarkers of advanced fibrosis in the evaluation of non-alcoholic fatty liver disease”

**DOI:** 10.1093/gastro/gou064

**Published:** 2014-11-03

**Authors:** Huseyin Kayadibi, Erdim Sertoglu, Metin Uyanik

**Affiliations:** ^1^Biochemistry Laboratory, Adana Military Hospital, Adana, Turkey, ^2^Biochemistry Laboratory, Ankara Mevki Military Hospital, Anittepe Dispensary, Ankara, Turkey and ^3^Biochemistry Laboratory, Corlu Military Hospital, Tekirdag, Turkey

Dear Editor,

We read with great interest the recently published article by Tapper *et al.* [1]. In this study, the authors' intention was to describe the clinical predictors of advanced histology in a tertiary referral centre population, and to describe the rate of advanced histology in patients with ‘normal’ ultrasound findings in a cohort of liver-biopsy-proven, non-alcoholic fatty liver disease (NAFLD) or non-alcoholic steatohepatitis (NASH) patients. In conclusion, aspartate aminotransferase-to-platelet ratio (APRI) greater than 1.0 was found to be the most significant predictor of advanced fibrosis. Additionally, 20% and 16.7% of patients without ultrasound-detected steatosis had advanced fibrosis and active NASH, respectively. In accordance with these findings, the authors suggested that patients with suspected NAFLD should routinely be evaluated for advanced liver disease, including non-invasive indices of fibrosis such as APRI, and that serious consideration should be given to liver biopsy. However, we would like to share our thoughts and contributions to the original study.

First, although liver biopsy is still accepted as the ‘gold standard’ method in diagnosis and follow-up of patients with advanced fibrosis, many studies have been performed on indirect, non-invasive, serum biochemical markers that can be used in place of liver biopsy because of its serious complications. APRI, Forns index, FIB-4, AST-to-ALT ratio, age-to-platelet index, hepascore, fibrotest, fibroindex, fibrometer and NASH fibrosis scores are the most commonly used of these tests. Among these tests, APRI and fibrotest have the highest diagnostic accuracy for liver fibrosis (AUCs are 0.94 and 0.97, respectively), therefore, following diagnosis by ultrasound, assessment of liver fibrosis in patients with NAFLD will reduce the risk of performing unnecessary biopsy.

Second, cut-off values of 1.5 and 2.0 were usually used for APRI, to predict significant fibrosis and cirrhosis, respectively. In the original study, 1.0 has been accepted as predicting both significant and advanced fibrosis. According to previous studies, 1.5 should be selected for significant liver fibrosis, and a cut-off point of more than 1.5 and less than 2.0 should be selected for advanced fibrosis [2]. Sensitivity of the 1.0 cut-off point was very low: only about 30%. Therefore, the selected cut-off point for APRI should be more than 1.0 to increase the sensitivity and negative predictive value.

In conclusion, due to probable liver fibrosis, patients with NAFLD should initially be evaluated using non-invasive liver fibrosis indices, adopting the previously established cut-off points. 

**Conflict of interest:** none declared.
